# Vegetarian Diets and Eating Disorders in Adolescents and Young Adults: A Systematic Review

**DOI:** 10.3390/children8010012

**Published:** 2020-12-28

**Authors:** Theodoros N. Sergentanis, Maria-Eleni Chelmi, Andreas Liampas, Chrysanthi-Maria Yfanti, Eleni Panagouli, Elpis Vlachopapadopoulou, Stefanos Michalacos, Flora Bacopoulou, Theodora Psaltopoulou, Artemis Tsitsika

**Affiliations:** 1 MSc Program “Strategies of Developmental and Adolescent Health”, 2nd Department of Pediatrics, “P. & A. Kyriakou” Children’s Hospital, School of Medicine, National and Kapodistrian University of Athens, 115 27 Athens, Greece; tsergentanis@yahoo.gr (T.N.S.); mchelmi@med.uoa.gr (M.-E.C.); chrysanthiyfanti@gmail.com (C.-M.Y.); elenpana@med.uoa.gr (E.P.); tpsaltop@med.uoa.gr (T.P.); 2 Department of Clinical Therapeutics, “Alexandra” Hospital, School of Medicine, National and Kapodistrian University of Athens, 115 28 Athens, Greece; 3 Clinical Psychopathology, University of Macedonia, 546 36 Thessaloniki, Greece; 4 Medical School, Department of Neurology, University of Cyprus, 1678 Nicosia, Cyprus; liampasand@gmail.com; 5 Department of Endocrinology-Growth and Development, “P. & A. Kyriakou” Children’s Hospital, 115 27 Athens, Greece; elpis.vl@gmail.com (E.V.); stmichalakos@gmail.com (S.M.); 6 Center for Adolescent Medicine and UNESCO Chair Adolescent Health Care, First Department of Pediatrics, “Agia Sophia” Children’s Hospital, School of Medicine, National and Kapodistrian University of Athens, 115 27 Athens, Greece; bacopouf@hotmail.com

**Keywords:** vegetarianism, vegetarian diets, eating disorders, mental health, adolescents, young adults

## Abstract

Background: Eating disorders are more common among adolescents and young adults. An increase in the rates of these disorders has been reported during the last years. Meanwhile, vegetarianism is becoming more popular in these age groups. The purpose of the present paper is to evaluate the association between eating disorders and vegetarian diets in adolescents and young adults. Methods: Systematic review of related articles published in PubMed, PsycInfo and Google Scholar up to 30 May 2019. Results: A total of 20 studies (14,391 subjects) were deemed eligible for this systematic review. The majority of the studies reported significant correlations between vegetarianism and eating disorders. However, due to the cross-sectional design, a causal link between eating disorders and vegetarian status cannot be established. Conclusions: Vegetarianism seems to be associated with eating disorders. Longitudinal studies are needed to establish temporal patterns between vegetarianism and the emergence of disordered eating.

## 1. Introduction

A vegetarian diet excludes meat, seafood and products containing both, but may include eggs and dairy [1]. Vegan diet is an extreme express of vegetarianism, excluding all animal products, namely everything based on animal origin such as additive fats [2]. Lately, there is an increasing movement toward vegetarian diets in different population groups, including adolescents (11–21 years) and young adults (up to 24 years), either due to personal initiative, ethical reasons or social factors [3]. In the United States, it has been estimated that 32% of adolescents between 8 and 18 years declare to have at least one vegetarian meal per week, while 4% of this age group is reported to completely follow vegetarian diet [3]. Concerning adults (>18 years) in the United States, the relevant rates are smaller, as 3.3% of them are recorded as vegetarians, half of them being vegans [3].

Elimination of animal products from the daily diet could potentially affect health especially that of adolescents whose development has not been yet complete. Although there is a correlation between vegetarian diet and health-related benefits, for instance regarding the decreased risk of cardiovascular disease [4,5], there is a large controversy about the effects of vegan diet on mental health [6].

Eating disorders (anorexia nervosa, bulimia nervosa and Eating Disorder Not Otherwise Specified—SCID [7]) are usually associated with restricted diets and are more commonly observed in adolescents and young adults. Vegetarianism has been increasingly considered as a way of weight control, as the diet is based in reduced animal fats [7]. In cases of eating disorders, the disorder itself leads to exclusion or restriction of specific products and different confounders and causes should be examined. According to the available literature, 45 to 54% of adolescents and young adults with anorexia nervosa followed some form of a vegetarian diet, especially females [7,8]. Additionally, in some cases (about 6%), patients have reported that they were vegetarians several years prior to the onset of their eating disorder [7]. It has been suggested that, when subjects with a suspected or diagnosed eating disorder follow a vegetarian diet, health care professionals should worry that this behavior may be used as a socially acceptable way to legitimize food avoidance [7] and avoid certain eating situations [8].

In light of the above, the aim of this systematic review was to investigate the correlation between vegetarian and vegan diets and eating disorders in adolescents and young adults.

## 2. Materials and Methods

### 2.1. Literature Search Strategy

A systematic literature search was performed on 30 May 2019 in three different databases (PubMeD, PsycInfo and Google Scholar). For the search, various terms were combined, namely vegetarian OR vegan OR vegans OR veganism OR vegetarian OR vegetarianism OR semi-vegetarian OR flexitarian OR lacto-ovo-vegetarian OR lacto-vegetarian OR lactovegetarian OR ovo-vegetarian OR fruitarian OR flexitarian, with “adolescents” OR “teenagers” OR “young adults” OR puberty OR youth OR “young adult” OR “young adults” OR “young adulthood” and anorexia OR bulimia OR “eating disorder”. The reference lists of eligible papers and relevant reviews were also meticulously searched in order to include further studies reporting on vegan diet and eating disorders.

### 2.2. Inclusion Criteria

The eligibility criteria were based on the PICOS (Participants, Intervention, Comparison, Outcomes, Study design) acronym. Articles eligible to be included in this review were required to meet the following criteria:

1. Studies had to report on adolescents or young adults (up to 30 years of age) who follow vegetarian or vegan diet. Studies could be purely based on adolescents and young adults or include a subgroup of young adults with admixture of older individuals. At any case, the results of these two subcategories of studies were presented separately.
2. Studies had to provide data about the correlation between vegetarian/vegan diet and eating disorders.
3. Any strategy to diagnose eating disorders was deemed eligible.
4. Prospective cohorts/cross-sectional/case-control studies were included.
5. The article was written in English language.
6. There was no restriction in publication year.

### 2.3. Exclusion Criteria

Articles meeting the following criteria were excluded from the review:

1. Case reports
2. Review articles and medical hypotheses
3. Animal studies
4. Papers referring to subjects with low-meat consumption (i.e., not vegetarians).
5. Studies not declaring age groups

All article abstracts were screened by authors working in pairs in a blinded fashion. Those found not complying with the inclusion criteria were removed and any controversies were dealt with consensus in a meeting, in which the abstracts were reviewed.

### 2.4. Quality Assessment of Included Studies

All studies were rated with the Newcastle–Ottawa scale, adapted for assessing the quality of non-randomized cross-sectional studies. This scale allocates a maximum of 10 stars evaluating selection (representativeness, sample size, nonrespondents and ascertainment of exposure), comparability and outcome (assessment, statistical test) [9].

### 2.5. Data Collection Process

Data were extracted from each study in a structured coding scheme using Excel and included type of study, population size, gender and age distribution, population type (exclusively adolescents or young adults or a mix of them), confounders, definitions of eating habits, definitions of eating disorders and bias assessment. Data were retrieved by authors in pairs and team consensus was ensured.

### 2.6. Compliance with Ethics Guidelines

This article is based on previously conducted studies. The study is performed in accordance with the Preferred Reporting Items for Systematic Reviews and Meta-Analysis (PRISMA) guidelines [10].

## 3. Results

### Study Characteristics

**Figure 1:**
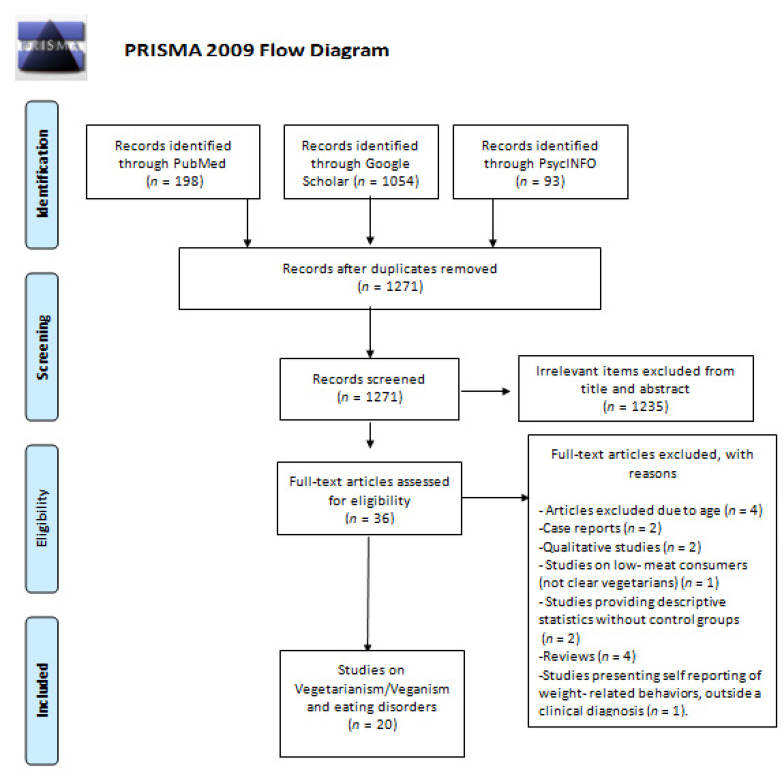
Successive steps in the selection of studies—PRISMA Flow Diagram.

The literature search produced a total of 1271 results, after removal of duplicates; of them 1235 were deemed irrelevant from title and abstract, whereas 36 were evaluated in full-text. Among the latter, 16 were excluded due to various reasons and ultimately, a total of 20 studies (14.391 subjects) published between 1986 and 2019 were included in the present systematic review [7,11,12,13,14,15,16,17,18,19,20,21,22,23,24,25,26,27,28,29]. The PRISMA flowchart describing the successive steps in the selection of studies is presented in Figure 1. The general characteristics of the studies are presented in Table 1. Table 1 also presents the summary findings of the quality assessment (risk of bias) for the included studies; in the majority, the quality of studies was rated as low.

The majority of included studies reported a positive association between vegetarianism/veganism and eating disorders. Specifically, Bardone-Cone et al. observed that individuals with a history of eating disorder were more likely to have ever followed a vegetarian diet compared to healthy controls [7]; similarly, Zuromski et al. recorded a higher prevalence of vegetarianism amongst women with clinical diagnosis of eating pathology [16]. Likewise, in a questionnaire survey by McLean et al., female students with vegetarian dietary restraints were more likely to have a history of eating disorders [19]. In a representative community survey, Michalak et al. confirmed the association between vegetarianism and eating disorders [17]. Accordingly, a positive association between being a vegetarian and diagnosis [13] or indication (according to EAT-26 [12,15,20,21] and EAT-40 scores) [14] of an eating disorder has been reported by other researchers.

Barrack et al. observed that, despite a correlation in the univariate analysis, the association between vegetarian status and eating disorder examination scores did not persist at the multivariate analysis [18]. Notably, Timko et al. reported a trend linking semi-vegetarianism with highest levels of restraint [11].

On the other hand, a correlation between vegetarianism and eating disorders was not documented in a few studies [26,28,29], whereas an inverse association between veganism and pathological attitudes towards food was reported [27].

Regarding prognosis and features of eating disorders (lower panels of Table 1), Kadambari et al. [22] showed that vegetarian anorectics were more frequently abstainers, hyperactive, consumed large quantities of non-calorific fluid and presented higher fear of fatness versus non vegetarian anorectics. In a retrospective study, O’ Connor et al. found that, as a rule, the avoidance of red meat did not predate anorexia nervosa [23]. Hansson et al. discovered that vegetarianism was more frequently present in recovered or current patients with anorexia nervosa [24]. Interestingly, Yackobovitch-Gavan et al. showed that vegetarianism (past and present) correlated with non-remission of anorexia nervosa [25].

## 4. Discussion

The present systematic review is an effort to investigate the association between different types of vegetarian diet and eating disorders in adolescents and young adults. As the literature research revealed, there seems to be a correlation between vegetarianism and the presence of eating disorders [7,12,13,14,15,16,19,21] and particularly anorexia nervosa [22,23,24]. Especially for adolescents, vegetarianism and unhealthy and extreme behaviors on weight control are reported to be interconnected [13]. Females seem to be more prompt to such associations [16,17,19,20]. However, there were also studies reporting no correlation between eating disorders and vegetarianism [26,28,29].

Commenting on potential causal associations, numerous individuals with disordered eating habits and a history of vegetarianism report that the adoption of a vegetarian diet followed their disorder [7,8,17,23,30]. It seems therefore that, in a patient with an eating disorder and vegetarianism, there is a high likelihood that vegetarianism could represent a mode of restriction in eating habits, as a part of their eating disorder pathology. Perpetuation of pathology cannot be ruled out, in a vicious circle where restriction begets restriction. At any case, there is need for future prospective studies to shed light onto the aspect of temporality that is corollary for the establishment of any etiological associations.

Individuals with eating disorders might become vegetarians as a means to control weight, as a strategy of food avoidance, but also for non-weight reasons [7]. From an eating disorders perspective, subjects who are prompt to follow vegetarianism for mainly non-weight reasons (e.g., ethics, religion) could at first seem less alarming than subjects with weight-loss motives [13,18,19] and body shape worries [12], however bias in reporting the actual reasons is a concern. Specifically, in view of the stigma of eating disorders, the disclosed reason for following vegetarianism may sometimes be hard to identify in clinical practice. For a patient with an eating disorder, declaring to be a vegetarian for animal rights reasons would seem more socially acceptable and less uncomfortable than the explicit disclosure of weight loss as a motive. Social acceptability bias in this instance would denote that an eating disorder patient would purportedly prioritize animal rights/ethics when in reality the behavior could be driven by a desire to restrict.

The prevalence of reasons underlying choices of vegetarianism differ between studies. Klopp et al. [14] highlighted health/nutrition (37.5%) as the most common reason for vegetarianism, followed by weight control (18.8%) and animal ethics (14.6%). However, Bas et al. reported another context, with the most common reason being taste preferences (58.1%), followed by healthier diet (19.4%) and weight control only in 9.6% of the sample [12]. Other mechanisms have also been postulated, such as that the life experience of a mental health disorder may “sensitize” the patient towards the suffering of other beings and animals (thus eliciting vegetarianism or veganism), whereas presence of common variables underlying both vegetarianism and eating disorders (such as perfectionism, high levels of responsibility, social values) may provide another alternative explanation [17]. On the other hand, being vegetarian has been associated with positive qualities, including morality, empathy and being self-sacrificial for the greater good [31]. Concerning motivations, veganism is an entity distinct from vegetarianism, as it reflects a self-defining way of life, putting in practice ethical and moral beliefs that strongly oppose processes of treating animals in the food and animal products industry [29].

Regarding subgroups in the spectrum of semi-vegetarianism, vegetarianism and veganism, Timko et al. highlighted that semi-vegetarians had a trend towards a more restrained eating pattern [11]. Further studies are needed to differentiate between vegans, vegetarians, and semi-vegetarians.

In view of the association between vegetarianism and eating disorders, individuals who adopt a vegetarian diet should be closely examined regarding their general eating attitudes/behaviors by clinicians, to evaluate the presence of extreme weight control attitudes [13,18,19] and eating disorder chronicity [7,23,32].

The findings of the present systematic review can be inscribed into a wider perspective, regarding adverse aspects of vegetarianism in terms of mental health. A meta-analysis on 17.809 individuals, published in 2020, highlighted that vegan or vegetarian diets were associated with a higher risk of depression but with lower anxiety scores, although the synthesized studies were of low quality [33]. However, another meta-analysis, also published in 2020, indicated that there was no association between consumption of a vegetarian diet and depression or anxiety [34]. Such complex interplays between vegetarianism, eating disorders and depression would seem addressing in future studies. Eating pathology has been longitudinally associated with depression in a meta-analysis of 42 studies, that highlighted the need to identify factors that are etiological to the development of both conditions [35]. The debate regarding vegetarian diets and any effects upon mental health remains vivid and open.

Our results should be interpreted with some caution given the limitations of the synthesized studies. Firstly, most studies were of a cross-sectional design, thus cannot clarify if there is a causal relationship between vegetarianism and disorder eating. Secondly, quality ratings were low, as evidences by the Newcastle-Ottawa scale. The majority of studies were based on population-based samples, except for a few ones on clinical samples compared versus other groups [7,16,22,23,24,25]. Furthermore, a great variety of questionnaires was used. Other limitations include the lack of differentiation of eating disorder diagnoses, the scarcity of subgroup data about bulimia the small sample size in some studies, the variability in the definition of vegetarianism, and the lack of a validated measure for assessing aspects of vegetarianism or veganism. Although there was no limitation about the cultures on which studies were based, the fact that only studies in English were included in this systematic review may have actually limited synthesized evidence mainly into countries with a Western culture and lifestyle. There was paucity of data regarding subgroups of vegetarian diets, such as flexitarian, lacto-ovo-vegetarian, lacto-vegetarian, ovo-vegetarian, fruitarian and flexitarian diets that might exhibit distinct features.

## 5. Conclusions

In conclusion, this systematic review highlights a potential association between vegetarianism and eating disorders. Future research should focus on multi-center prospective studies in order to shed light on temporal patterns in the relationship between vegetarianism and disordered eating, although such studies would be practically demanding.

## Figures and Tables

**Table 1:** Characteristics of included studies. Upper panels present studies encompassing exclusively on adolescents and young adults; middle panels present studies on youth, but with admixture with older individuals; lower panels present studies on prognosis/features of eating disorders.

Author (Year)	Region, Country	Study Period	Study Design	Sample Size	Number of Vegetarians	Percentage of Males	Mean Age (SD)	Age Range	Study Population	Definition of Dietary Factor	Definition of Eating Disorders	Main Findings of the Study	Potential Cofounding Factors Assessed	NOS Quality Rating
**Studies exclusively on adolescents and young adults**														
Bardone-Cone 2012 [7]	USA (white 90%)	2007–2008	Case-control	160	56	0	23.61 (4.80)	16 and older	Patients seen at the University of Missouri Patients and Adolescent Specialty Clinic and university students as an additional control sample.	Self-reporting for having ever been vegetarian.	Patients seen at the University of Missouri Patients and Adolescent Specialty Clinic. clinical diagnosis (Structured Clinical Interview for DSM--IV)	Individuals with an eating disorder history were considerably more likely to ever have been vegetarian (52% vs 12%; *p* < 0.001).	None	2/10
Barrack 2019 [18]	USA (Hispanic 57%)	NR	Cross-sectional	106	5	37	18	NR	Students	Vegetarian status not explicitly defined.	Eating Disorder Examination Questionnaire (EDE-Q)	While following a vegetarian diet was associated with higher EDE-Q scores in the univariate logistic regression analysis (OR = 9.8, 95%CI: 1.0–91.4, *p* < 0.05), vegetarian status did not persist as a significant independent predictor in the multivariate regression model.	None	3/10
Bas 2005 [12]	Turkey	NR	Cross-sectional	1205	31	50	21.5 (1.9)	17–21	College students	Vegetarian status not explicitly defined.	Eating Attitude Test 26 (EAT 26)	Vegetarians presented with higher EAT-26 scores in males (17.25 ± 11.18 vs 9.38 ± 6.60, *p* = 0.019) and females (22.04 ± 13.62 vs. 11.38 ± 8.28, *p* < 0.001. Similar differences were noted in dieting and oral control, but not bulimia/preoccupation subscales.	Subgrouping on sex	4/10
Fatima 2018 [26]	Saudi Arabia	NA	Cross-sectional	120	12	0	20.7	18–23	College students	Vegetarian status not explicitly defined.	Eating Attitude Test 26 (EAT 26)	Vegetarianism in college women was not associated with disordered eating attitudes (EAT26), as the score in vegetarians (15.42 ± 11.57) did not differ versus non-vegetarians (16.07 ± 9.11), *p* = 0.83.	None	2/10
Fatima 2018 [20]	Saudi Arabia	From October 2017 to January, 2018	Cross-sectional	314	21	17.1	NR	15–19	Adolescents students	Vegetarian status not explicitly defined.	Eating Attitude Test 26 (EAT 26)	Vegetarian adolescent girls had higher scores in the dieting (*p* < 0.01) and oral control subscales (*p* = 0.01), total EAT 26 scores (20.67 ± 13.21 vs. 13.21 ± 9.07, *p* < 0.01) than non-vegetarians.	None	2/10
Fisak 2006 [28]	USA (Caucasians 70%)	NR	Cross-sectional	256	52	0	21.07 (3.75)	NR	Undergraduate students	Vegetarians	EAT-40, EDI	No significant differences were found between vegetarians and non-vegetarians in measures of eating pathology, such as EAT-40 (67.22 ± 30.52 vs. 57.86 ± 25.84, t = 1.85), EDI-DT (17.91 ± 11.29 vs. 15.51 ± 9.73, t = 1.26) and EDI-B scores 8.92 ± 7.48 vs. 6.85 ± 6.81, t = 1.59).	None	4/10
Klopp 2003 [14]	USA (white 82%)	NR	Cross-sectional	143	30	0	19 (1)	NR	Students	Self report	Eating Attitudes Test (EAT-40) >30.	The percentage of subjects with EAT-40 score > 30 was higher in vegetarians (36.7%) vs. non-vegetarians (8.8%), *p* = 0.0001.	None	5/10
Lindeman 2000 [21]	Finland	NR	Cross-sectional	118	15	0	16.44	16–18	High school students.	Vegetarians and non-vegetarians based on food choices questionnaire (not further defined)	EAT-26 (eating attitudes test) >20	20% of the vegetarians scored >20 in EAT-26, vs. only 3.9% in non-vegetarians	None	4/10
McLean 2003 [19]	Canada	1997	Cross-sectional	596	47	0	21.5(3.9)	NR	Students	Self-report	Self report about having ever been diagnosed or treated for an eating disorder	A higher percentage of the vegetarian participants had a history of eating disorders (17.1% vs. 3.1%, X^2^ = 17.9, *p* < 0.001). Vegetarian women had also lower self esteem.	None	4/10
Perry 2001 [13]	USA (white 48%)	1998–1999	Cross-sectional	4746	262	52	14.9	11 to 18	School students	Self report on a survey.	Previous diagnosis by a physician as having an eating disorder.	A positive association between being a vegetarian and diagnosis of an eating disorder was reported (adjusted OR = 2.72, 95%CI: 1.71–4.34)	Gender and race/ethnicity	6/10
Timko 2012 [11]	USA (Caucasians 80%)	NR	Cross-sectional	486	146	23	24.94 (9.54)	The majority (69.50%, *n* = 338) of participants were between 18 and 25 years old,	Students, internet, local stores	FFQ	Eating attitudes test-26 (EAT-26)	Semi-vegetarians reported the highest levels of restraint, however no significant differences were noted between groups in EAT-26 scores (9.22 ± 13.39 for vegans, 10.08 ± 11.64 for vegetarians, 11.81 ± 12,23 for semi-vegetarians and 7.98 ± 8.91 for omnivores), *p* = 0.052, Kruskal-Wallis test.	None	4/10
Trautmann 2008 [15]	USA (Caucasians 86.1%)	NR	Cross-sectional	330	30	28.8	18	NR	College students	Self report	EAT-26 (Eating Attitudes Test) >20	The mean EAT-26 score of vegetarians (M = 13.21) was significantly greater than non-vegetarians (M = 8.38, *p* = 0.006).	None	4/10
**Studies on adolescents and young adults, with admixture with older individuals**														
Heiss 2017 [27]	NR (internet users with fluency in English language)	NR	Cross-sectional	557	357	19.6	30.59 (12.61)	NR	Internet survey targeting mostly vegans	Self-report	Eating Disorder Examination-Questionnaire (EDE-Q) global score and subscales; Eating Disorder Inventory-Drive for Thinness (EDI-DT); Binge eating scale (BES).	Vegans endorsed less pathological attitudes and behaviors towards food in terms of EDE-Q-Global (1.82 ± 1.22 vs. 2.23 ± 1.38, *p* < 0.01) and subscales, EDI-DT (3.90 ± 5.43 vs. 5.27 ± 6.13, *p* = 0.05) and similar in terms of BES (2.81 ± 4.19 vs. 3.32 ±4.38, *p* = 0.19) versus omnivores.	None	4/10
Michalak 2012 [17]	Germany	1998/ 1999	Cross-sectional	4181	77	55	40	18–65	National survey	Self-report, food frequency	Computer-assisted version of the Munich Composite International Diagnostic Interview (M-CIDI)	Vegetarians displayed elevated prevalence rates for eating disorders 5.6% in completely vegetarians vs 1.2% in a non-vegetarian matched sample.	Matching on sex, age, educational level, size of the community, marital status	6/10
Norwood 2018 [29]	Australia (79% Caucasian)		Cross-sectional	393	176	17	29.38 (13.12).	17–74	Community and students	Self reporting of vegan/vegetarian/paleo/gluten free/weight loss and unrestricted diet	5-item version of the Eating Disorder Inventory, emotional eating subscale from the Dutch Eating Behavior Questionnaire, Dieting Intentions Scale, Trait General Food Cravings Questionnaire, Brief Self-Control Scale, 21-item Depression, Anxiety and Stress Scale, Positive and Negative Affect Scale,	People following vegetarian diets (mean = 1.04) did not significantly differ from the non-restricted comparison group (mean = 1.10) regarding eating disorders.	None	4/10
Zuromski 2015 [16]	USA (white/European origin 90%)	NR	Cross-sectional	142	29	0	21.3	NR	Clinical: female patients receiving residential treatment at an eating disorder center vs. nonclinical group, denying any lifetime eating pathology.	Lifetime vegetarian in self-report	Clinical: female patients receiving residential treatment at an eating disorder center	The prevalence of lifetime vegetarianism was lowest in the nonclinical group (6.8%) and highest in the clinical group of eating pathology (34.8%), *p* < 0.05. Intermediate prevalence (17.6%) was noted in the subclinical group,	None	3/10
**Studies on Prognosis/features of Eating Disorders**														
Hansson 2011 [24]	Sweden	August 2001–July 2002	Cross-sectional	131	NR	0	26.2 (6.5)	15–50	Eating disorders patients and controls	Vegetarians or omnivores based on food preferences questionnaire	Clinical diagnosis of anorexia or bulimia	Vegetarianism was more prevalent in recovered or current anorexic patients.	None	4/10
Kadambari 1986 [22]	UK (London area)	1968–1979	Cross-sectional	200	77	11.5	23	NR	Eating disorders patients	Self-report (vegetarianism was defined as the exclusion from the diet of food obtained by killing animals)	Clinical diagnosis	Vegetarianism characterized 45% of the anorectic population. Vegetarian anorectics were more likely to be abstainers, hyperactive and showed greater fear of fatness than nonvegetarian anorectics	None	2/10
O’Connor 1987 [23]	Australia	1982–1986	Cross-sectional	116	64	3.5	23 (6.7)	NR	Anorexia nervosa patients	Avoidance of red meat	Clinical diagnosis	54.3% patients were avoiding red meat. However, in only four (6.3%) of these did meat avoidance predate the onset of AN. The remaining 59 patients were termed pseudovegetarians and were associated with longer presence of anorexia nervosa.	None	2/10
Yackobovitch-Gavan 2009 [25]	Israel	1987–1999	Cross-sectional	91	NR	0	22–23	NR	AN patients	Vegetarians and non-vegetarians based on the Eating Disorders Family History Interview	Clinical diagnosis of AN	Vegetarianism (past and present) was correlated to non-remission of AN (OR = 0.095, 95%, CI: 0.011–0.789)	None	3/10

AN: anorexia nervosa; BES: Binge Eating Scale; CI: confidence interval; EAT: Eating Attitudes Test; EDI: Eating Disorder Inventory; EDI-DT: Eating Disorder Inventory “Drive for Thinness” subscale; EDE-Q: Eating Disorder Examination-Questionnaire; NOS: Newcastle-Ottawa Scale for the assessment of quality of studies; NR: not reported; OR: odds ratio.3.2. Results of individual studies.

## Data Availability

Data is contained within the article.
